# Exploring the Relationship Between Substance Use and Allostatic Load in a Treatment/Research Cohort and in a US Probability Sample (NHANES 2009–2016)

**DOI:** 10.3389/fpsyt.2021.630195

**Published:** 2021-08-02

**Authors:** Jeffrey M. Rogers, David H. Epstein, Karran Phillips, Justin C. Strickland, Kenzie L. Preston

**Affiliations:** ^1^Behavioral Pharmacology and Therapeutics Research Branch, National Institute on Drug Abuse, National Institutes of Health, Baltimore, MD, United States; ^2^Department of Psychiatry and Behavioral Sciences, Johns Hopkins University School of Medicine, Baltimore, MD, United States

**Keywords:** allostasis and allostatic load, substance use disorder, addiction, stress, chronic stress

## Abstract

Allostatic load, an operationalization for cumulative strain on physiology from adaptation (allostasis) to stress over a lifetime, can manifest as damage to cardiovascular, neuroendocrine, and metabolic systems. The concept of allostatic load may be particularly useful in research on substance-use disorders (SUDs) because SUD researchers have sought to better understand the relationship between chronic stressors and drug use. Theoretical models hold that SUDs can be conceptualized as a spiral toward a state of *persistent* allostasis (i.e., allostasis so persistent as to represent homeostasis at a new, unhealthy set point). Regardless of the extent to which those models are accurate, increased allostatic load could be a mechanism by which frequent drug administration increases risk for adverse outcomes. We conducted two secondary analyses to evaluate allostatic load in the context of drug use, including alcohol use, in a locally recruited sample with a high proportion of illicit substance use (*N* = 752) and in a nationally representative sample from the NHANES 2009–2016. We hypothesized that after controlling for age and other potential confounds, people with longer histories of drug use would have higher allostatic-load scores. Multiple regression was used to predict allostatic load from participants' drug-use histories while controlling for known confounds. In the locally recruited sample, we found that longer lifetime use of cocaine or opioids was related to increased allostatic load. In NHANES 2009–2016, we found few or no such associations. Lengthy histories of problematic non-medical substance use may facilitate more rapid increases in allostatic load than aging alone, and, together with findings from previous investigations, this finding suggests increased risk for chronic disease.

## Introduction

Allostatic load refers to a state of accumulated physiological stress that increases the likelihood of morbidity. Allostasis, a term first used by Sterling and Eyer (1), refers to systems that when functioning normally, provide an adaptive response to environmental or psychological perturbations by modulating cardiovascular, metabolic, neuroendocrine, and immune mechanisms. However, when chronically activated or under-activated in response to unremitting perturbations, these physiological systems incur damage (2). Allostatic load theory was originally constructed to explain how chronic stress damages physiologic systems and accelerates aging, but allostatic load research has been extended to many topics related to chronic stress. There has been longstanding research interest in uncovering the ways in which both internal and external stressors affect initiation of, maintenance of, and relapse to substance-using behaviors, and, unsurprisingly, considerable theoretical work has linked the concept of allostatic damage with aspects of substance-use disorders (SUDs). Following shortly after one of the most influential allostatic load publications (2), an allostasis model of addiction/SUDs proposed by Koob and Le Moal (3) considered the downward spiraling of the brain's reward system in the context of allostatic load, whereby an individual's reward system moves further and further from its original homeostatic set point with each drug administration until arriving at a persistent allostatic state that manifests partly as addiction. In later work, the authors expanded upon this theory, providing preclinical and clinical evidence for a process whereby an allostatic state in brain reward and stress systems can increase addiction vulnerability (4).

Summarizing the current state of conceptual work linking allostasis theory and SUDs, there are two main hypotheses that are common among allostasis-addiction theories. First, stress is a key factor underlying the development and maintenance of SUDs, and second, repeated drug administration can negatively affect allostasis systems (5). Severe or chronic stress prior to initiation of substance use will increase the likelihood of an SUD, because already over-taxed allostasis systems may decompensate more rapidly, and those who develop an SUD may incur a more dramatic increase in allostatic load than they would with aging alone, independent of unhealthy behaviors that may accompany the SUD. Proposed mechanisms of this “accelerated aging” include cellular oxidation, excessive tissue inflammation, and stress-hormone dysregulation. These phenomena remain largely undetected in clinical substance-using populations (6), but there is some evidence for them with specific classes of drugs such as psychostimulants and opioids. Specifically, people with histories of cocaine-use disorder (CocUD) may present with delayed maturation of white matter, volume reductions in gray matter, telomere shortening, and reductions in some measures of cognitive functioning (7–11). People with histories of opioid-use disorder (OUD) may present with more rapidly developing arterial stiffness, suppressed activity in peripheral telomerase, and structural deterioration in prefrontal gray and white matter (12–14). For a comprehensive review of current evidence supporting an allostasis model of SUDs, see Fronk et al. (5).

These findings suggest that SUDs are accompanied by considerable physiologic dysregulation. But it remains unclear whether allostatic load, as measured in downstream cardiovascular, metabolic, and immune systems, differs as a function of drug-related stress. This would be an important finding because it would have health implications of its own, regardless of whether other aspects of allostasis theories of addiction are ultimately supported [As noted by psychologist Murray Sidman (15), “Good data are notoriously fickle. They change their allegiance from theory to theory, and even maintain their importance in the presence of no theory at all.”] To begin to clarify the data on allostatic load, we must first briefly review the methods with which allostatic load is typically operationalized.

There is no one set of measurements that is uniformly used to represent allostatic load. Typically, investigators assemble panels of physiological measurements and assays to represent multiple systems involved in or affected by allostasis (16–20). The panels are then scored according to any of several summation procedures, and the resultant score is treated as the allostatic load (21). The most common scoring method is measure-by-measure dichotomization, with cutoff points set within the distribution of each measure. Typically, either the highest or lowest quartile represents the greatest likelihood of morbidity and is scored 1, while the other quartiles are scored 0; and the dichotomized values for all measures are then summed to generate each person's allostatic load score (21). This risk-dichotomization scoring has some major drawbacks: designating arbitrary cutoff points depends largely on normality within measurement distributions, and not all measurements carry the same predictive weight in unique populations.

Some of the drawbacks of dichotomization can be avoided by using summed z-scores scaled by canonical weights in canonical correlation analysis [CCA, (16)]. Canonical correlation analysis assigns a unique variable-importance weight to each measure by assessing the overall correlation between the full set of measures (X) and a group of physical and cognitive functioning measures (Y) (22–24). The overall correlation contains a contribution from each measure in X, and these contributions, called latent associations, can be used to weight the value of each measure before summing all the measures to generate a numerical score for allostatic load. This technique avoids the issue of under- or over-assigning risk in a dichotomous fashion, as each score is a continuous product of weighted z-scores. Also, because canonical weights can themselves be conceptualized as variable-importance scores, scaling with canonical weights allows the most clinically relevant measures to have greater influence over the final allostatic-load score (16, 23).

Although canonical-weight scaling and risk dichotomization are substantially different scoring methods, and although measurement panels vary widely across studies, allostatic-load scores are consistently found to be associated with psychosocial and demographic variables, regardless of the methodology used. Examples of such associations with higher allostatic load (even after controlling for age) include adverse or traumatic life experiences (25–29), lower socioeconomic position (20, 30–34), African American race in the US (35–40), perceived stress (41–43), psychiatric disorders (44–48), and poor sleep quality (49–52). Even after controlling for socioeconomic and demographic factors, allostatic load has shown the robust ability to predict later functional decline and all-cause mortality (17, 53). Thus, allostatic load is useful not only for identifying risk at the demographic level, but also for understanding differing risks within demographic groups.

In previous research using CCA to scale allostatic-load measures, the set of comparison variables (Y) has reflected physiological and cognitive functioning, but not sociodemographic variables (16, 22, 23). This method has been criticized: Seplaki et al. (54) argued it can result in model overfit, meaning that the results are less generalizable to other samples. Because psychosocial and demographic variables have shown equally robust associations with allostatic load as measures of function, they may be equally effective in generating scaling weights. Scaling allostatic-load scores to known predictors, rather than measured physiologic outcomes that allostatic-load scores are often intended to predict, helps ensure that the scores will not be overfitted to person-specific outcomes within a particular sample. To evaluate overfitting, reference-set scaling variables can be included as covariates in downstream analyses, and sensitivity analysis can be conducted using a dichotomously scored allostatic-load measure *post-hoc*.

The purpose of the two secondary analyses reported here (as Study 1 and Study 2) was to determine whether previously established relationships among allostatic load, sociodemographic factors, sleep quality, and indices of psychological stress could be observed cross-sectionally in people with SUDs or histories of non-medical drug use, and whether participants' drug-use histories would predict allostatic load scores after controlling for relevant covariates. As reviewed above, evidence links psychological and physiological stress measures to the development and maintenance of SUDs in clinical samples, but there is less evidence for increased allostatic load in people with histories of non-medical drug use in the general population.

Study 1 was conducted in our own research clinic, using a panel of allostatic-load measures that included a primary mediator of allostasis and a proxy measure of stress-hormone release that we used to construct a continuous score for allostatic load. The Study 1 sample consisted of people with a high overall prevalence of, but substantial variability in, non-medical drug use and SUDs, strengths absent in many prior studies of drug use and allostatic load. In Study 2, we tried to replicate our results using data from a nationally representative sample. We chose the National Health and Nutrition Examination Survey (NHANES) 2009–2016 because the data were suitable (i.e., included biological measures) and were collected during the same time period as our own. NHANES assessments include information on non-medical use of drugs (including frequency and duration of use), but no information on symptoms of SUDs. Therefore, NHANES respondents who report use include people with and without SUDs, and an SUD diagnosis cannot be inferred from frequency and duration of use. We nonetheless expected that the NHANES data set, by dint of its size, would show some relationship between allostatic load and frequent, long-term non-medical use of psychoactive drugs, even if that relationship occurred only in the (unknowable) subset of respondents who had SUDs.

## Study 1 Method

### Clinical Study Sample

The clinical sample consisted of 752 adults participating in either of two studies we conducted at our outpatient treatment-research clinic in Baltimore, MD. The data from these studies were combined for the secondary analyses presented here.

One study (*N* = 150, 19.9% of the total sample) enrolled people seeking treatment for OUD and was primarily designed to use Ecological Momentary Assessment (EMA) via smartphone as a method of studying stress, craving, and drug use in daily life. Participants were treated with either methadone or buprenorphine for up to 22 weeks with the option for an 8-week taper (or help with transfer to a community clinic) following their participation. These participants were compensated at $20.00/h, not to exceed $100.00, for the laboratory session during which they completed the AL and baseline measures. The main exclusion criteria for this study were any history of DSM-IV psychotic disorder, bipolar disorder, current Major Depressive Disorder, physiological dependence on alcohol or any sedative-hypnotic, some medical illnesses (e.g., cirrhosis, nephrotic syndrome, thyroid disease, ischemic heart disease, epilepsy, panhypopituitarism, and adrenal insufficiency), or use of medications that, in the view of the investigators, would compromise participation in research (e.g., glucocorticoids, adrenal extract supplements, spironolactone, and pregnenolone).

The other study, which we refer to as the Health Outcomes by Neighborhood or HON study (*N* = 602, 80.1% of the total sample) enrolled any adult who consented, regardless of drug-use status, for a cross-sectional examination of the social and environmental factors that influence illicit drug initiation, addiction, and treatment seeking. Health Outcomes by Neighborhood study participants were given the same compensation for allostatic load and baseline measures as participants in the treatment study. Exclusion criteria for the HON study were minimal: we excluded only people who were deemed unable to provide informed consent or biologic samples.

In a combined dataset of the two cohorts, the mean age was 37.7 ± 11.2 years (range, 18–66); 472 (62.8%) participants were male, 558 (74.2%) identified as Black, 189 (24.6%) identified as White, 4 identified as Hispanic (<1%), and 1 identified as Asian. The five participants identifying as either Hispanic or Asian were coded as Black in subsequent analyses for a total 563 (74.9%). For participants who were enrolled in both studies (*N* = 52), we used only their data from the OUD study. We have published other data from the OUD study (55–61), and from the HON study (62). This work was supported by the NIDA Intramural Research Program.

### Allostatic-Load Measurement

In the HON study, participants were asked to begin fasting (except for water) at 10:00 p.m. the night before their 08:00 a.m. arrival at the clinic, for a fasting time of approximately 10 h. Participants provided a urine sample and breathalyzer upon arrival, and allostatic load measurements were postponed if participants were acutely intoxicated or had a breath alcohol content greater than zero. Breakfast was provided shortly after allostatic load specimen collection but prior to administration of stress and drug use questionnaires (DUG). Participants were typically given a short break prior to completing mental, physical, and social history questionnaires. Vital signs (blood pressure and heart rate) were taken following at least 30 min of rest with no caffeine. Seated systolic (SBp) and diastolic (DBp) blood pressure were measured three times, and the average of the second and third readings was used; the first was excluded to permit habituation to the cuff. Participants' height, weight, and waist and hip circumference were used to calculate the waist-to-hip ratio (WHR) and body mass index (BMI) variables. Waist circumference was operationalized as the narrowest point between the ribs and iliac crest; hip circumference was measured at the point where buttocks reached maximal circumference. For blood serum assays, participants had approximately 30cc (or approximately 2 tablespoons) of blood drawn. Following initial processing and de-identification at NIDA IRP, the samples were tested at a commercial laboratory. Analytes and analyte-derived measures were creatinine clearance (CRE), albumin (ALB), low-density lipoprotein (LDL), high-density lipoprotein (HDL), glycated hemoglobin (HbA1c), C-reactive protein (CrP), dehydroepiandrosterone sulfate (DHEAs), total cholesterol ratio (TCR), and triglyceride (TRI). Total cholesterol ratio was defined as the ratio of LDL to total serum cholesterol, a measure more indicative of cardiovascular health than LDL alone.

For participants in the OUD study, data collection differed only slightly. After consenting and enrolling in the study, participants began buprenorphine or methadone treatment and were stabilized on their dose for weeks one through three. The laboratory session took place during week two. Participants were fed a light breakfast and given their daily methadone or buprenorphine dose after the allostatic load measurements, but before questionnaire administration. Participants were allowed to complete the questionnaire packet on a separate date prior to study week four if they were unable during session one. Otherwise, methods did not differ from those of the HON study.

### Psychosocial, Drug Use, and Demographic Measures

Global psychological stress was assessed with the Perceived Stress scale (PSS) (63), a 14-item measure of self-rated stress levels and coping ability over the past month. Sleep quality was assessed with the 10-item Pittsburgh Sleep Quality Questionnaire (PSQI). Scores on the PSQI were summed according to published guidelines such that higher scores reflect poorer sleep; generally, scores greater than five are thought to indicate poor sleep quality (64). The 17-item Life Events Checklist (LEC) was used to assess exposure to highly salient traumatic events that are known to precipitate post-traumatic stress disorder (65). Life Events Checklist responses were scored to reflect the proximity of the respondent to the event as it occurred (i.e. “Happened to me” = 3, “Witnessed it” = 2, “Learned about it” = 1), and scores were summed into a single summary measure reflecting total lifetime exposure.

Drug use was assessed by the Addiction Severity Index (ASI), a structured interview that includes items on the number of lifetime years and past-month days for use of alcohol, heroin, methadone, other opioids, barbiturates, other sedatives, cocaine, amphetamines, cannabis, hallucinogens, and inhalants (66). Because responses in our sample were mostly limited to alcohol, non-medically used opioids, cocaine, and cannabis, those four drugs were the focus of our analyses. The proportions of participants self-reporting use of those drugs are listed in Table 1. Responses to the “heroin” and “other opioid” items on the ASI were collapsed into one “opioid” variable for regression analysis. We were unable to include tobacco use history in our analyses, as a substantial portion of participants did not complete a long-term retrospective measure of tobacco use. The ASI was administered as part of a screening measure prior to the first study visit. Screening data were also used to ascertain participant's age, race, sex, and attained education level. Descriptive data for all continuous measures are reported in Table 2.

**Table 1:** Proportions of participants reporting use for the four most common drugs on the Addiction Severity Index (ASI).

	**Opioids**	**Cocaine**	**Cannabis**	**Alcohol**
Total prevalence	0.42	0.34	0.51	0.47
>5 Years	0.31	0.20	0.28	0.23
>10 Years	0.22	0.12	0.13	0.14
>20 Years	0.12	0.04	0.03	0.07

**Table 2:** Means and standard deviations for psychosocial variables and individual allostatic load measures.

	**White**				**Black**			
	**Female (***N*** = 64)**		**Male (***N*** = 125)**		**Female (***N*** = 212)**		**Male (***N*** = 351)**	
	***M***	**σ**	***M***	**σ**	***M***	**σ**	***M***	**σ**
Age	35.80	10.90	36.00	10.60	35.30	11.30	40.20	11.00
Education years	12.50	2.86	12.60	2.78	12.70	3.16	12.40	2.75
Perceived stress	22.40	7.90	24.40	6.58	22.00	6.80	23.80	6.64
Pittsburgh sleep quality	6.27	4.52	6.38	3.67	6.51	3.61	6.45	3.65
Traumatic events—LEC	4.03	3.43	4.65	3.65	3.62	2.57	3.82	2.93
Opioid years	1.99	4.03	6.11	7.67	3.95	8.79	7.79	10.80
Cocaine years	1.48	4.68	3.03	5.95	2.53	6.18	4.38	7.42
Cannabis years	2.09	4.76	4.45	5.09	2.79	5.37	5.74	7.70
Alcohol years	4.09	7.80	5.40	7.34	2.29	6.20	5.68	9.13
Allostatic load	−3.67	7.42	−0.35	7.54	−0.68	7.05	1.20	7.39
Systolic blood pressure	111.00	13.30	118.00	15.00	114.00	12.30	122.00	14.60
Diastolic blood pressure	70.80	8.99	75.10	10.50	71.60	9.68	77.30	11.40
Waist–hip ratio	0.82	0.07	0.83	0.08	0.89	0.09	0.87	0.07
Body mass index	27.10	6.10	30.90	8.05	26.30	5.47	26.40	5.62
Glycosylated hemoglobin	5.32	0.68	5.52	0.44	5.37	0.38	5.62	0.76
C-reactive protein	0.50	0.43	0.57	0.51	0.52	0.67	0.48	0.66
Serum triglyceride	118.00	77.60	90.10	58.00	126.00	75.40	92.60	55.20
High density lipoprotein	53.30	17.40	57.70	18.70	46.10	11.50	55.30	16.50
Total cholesterol ratio	3.80	1.41	3.31	1.18	4.01	1.24	3.35	1.18
DHEA-S	137.00	89.70	140.00	81.90	204.00	112.00	198.00	116.00

### Analyses

The final panel of physiological measures for allostatic load included twelve items, three of which (BMI, WHR, and TCR) were computed from multiple measures. The distributions for each of the 12 were checked for symmetry and, apart from CrP, were log transformed (SBp, DBp, BMI, HbA1c, ALB, DHEA-s, TCR, HDL, TRI) or square root transformed (WHR, CRE).

We used CCA to assess latent linear relationships between the allostatic-load variable set (X) and the set of psychosocial and demographic variables (Y). Canonical correlation analysis generates canonical functions equal in number to the variables in the smaller of the two datasets (X and Y). In the calculation of each canonical function, the relationship between the two variable sets is maximized, but such that each successive function assumes none of the relationship used in the preceding function(s). Percent canonical relationship referenced below indicates the amount of possible information in the canonical space explained by the function in question. We generated a weight for each variable in the analysis, representing the size of that variable's contribution to the correlation. Then, following the method used by Karlamangla et al. (23), we used the canonical weights to scale z-scores for the allostatic-load measures, prior to summing them into a single continuous allostatic-load score. Log- or root-adjusted allostatic load scores were converted back to their raw value before computing z-scores and scaling. Of the 750 participants with allostatic-load data, 43 (5.7%) did not complete the PSS measure. These values were filled using multiple imputation and predictive mean matching via the *R* package {mice}.

Multiple linear regression was used to test the hypothesis that drug-use histories would predict allostatic load scores after controlling for sociodemographic factors (age, race, sex), sleep quality (PSQI), perceived stress (PSS), and adverse life events (LEC). Multicollinearity checks were performed using pairwise correlation comparisons, chi-square tests, and Farrar-Glauber tests.

## Study 1 Results

Here, seven functions (canonical variables, CVs) were generated, four of which were deemed significant by Rao's *F* approximation. The approximated *F*-statistic becomes less precise as the number of canonical functions increases; an approximated *F*-value would be exact for a single generated function. CV1 ($R_{c}^{2}$ = 0.48, *F* = 14.71, *p* < 0.01) accounted for 47.01% of the total canonical relationship and 77.4% of the variance shared between the two variable sets (1 – Λ_CV1_ = 1 – 0.226). CV2 ($R_{c}^{2}$ = 0.42, *F* = 10.03, *p* < 0.01) accounted for 37.41% of the canonical relationship and 56.6% of the variance shared between the two variable sets (Λ_CV2_ = 0.434). CV3 ($R_{c}^{2}$ = 0.19, *F* = 4.28, *p* < 0.01) accounted for 11.65% of the canonical relationship and 24.6% of the variance shared between the two variable sets (Λ_CV3_ = 0.754). CV4 ($R_{c}^{2}$ = 0.03, *F* = 1.57, *p* = 0.02) accounted for 1.92% of the canonical relationship and 7.3% of the variance between the two variable sets (Λ_CV4_ = 0.927). Functions 4 through 7 combined accounted for <3.0% of the canonical relationship.

We chose to use only CV1 weights (Table 3) for the following reasons. First, CV1 always represents the best possible linear relationship between the two variable sets; second, though the *R*^2^-values do not differ substantially between CV1 and CV2, CV1 explains approximately 20% more of the variance between the variable sets; third, in published analyses, only the first canonical variable was used to scale allostatic load scores, though this may have been due to non-significant subsequent CVs (23, 24).

**Table 3:** Canonical coefficients and structural correlations generated by CCA.

	**Canonical coefficients**	**Structural correlations**
**CV1 (X)**		
Systolic blood pressure	0.612	0.328
Diastolic blood pressure	2.639	0.494
Waist–hip ratio	5.016	0.341
Body mass index	0.440	0.163
HbA1c	2.170	0.427
C-reactive protein	0.136	0.080
Triglyceride	0.174	0.175
High density lipoprotein	1.713	0.167
Total cholesterol ratio	1.207	0.045
Creatinine	0.015	0.098
Albumin	2.432	0.446
DHEAs	0.902	0.770
**CV1 (Y)**		
Age	0.085	0.924
Black race	0.304	0.231
Male sex	0.717	0.185
Education	0.001	0.032
PSS	0.015	0.150
PSQI	0.013	0.238
LEC	0.001	0.081

We used multiple linear regression to test the hypothesis that years of regular drug use would predict higher allostatic load while controlling for other likely predictors. The overall model fit was significant [*F*_(11,739)_ = 40.15, *p* < 0.01] with adjusted *R*^2^ = 0.37. Greater allostatic load was associated with greater age (β = 0.45, 95%*CI* [0.38, 0.52]), Black race (β = 0.15, 95%*CI* [0.02, 0.28]), greater perceived stress (β = 0.08, 95%*CI* [0.01, 0.14]), lengthier opioid use histories (β = 0.09, 95%*CI* [0.02, 0.16]), and lengthier cocaine use histories (β = 0.12, 95%*CI* [0.05, 0.19]). The variance inflation factors (VIFs) for all covariates were less than or equal to 1.47, indicating that collinearity did not pose serious barriers to interpretation of the model. A full summary for the “years of regular drug use” model is presented in Table 4.

**Table 4:** Summary for a multiple linear regression that considers allostatic load as a function of years spent using drugs as reported on the addiction severity index.

	**Overall model test**								
	***R***	***R^2^***	***Adj R^2^***	***F***	***df1***	***df2***	***p***		
Drug years model	0.61	0.37	0.37	40.10	11	739	< 0.001		
						**95% CI**		**Collinearity**	
**Predictor**	***B***	***SE***	***t***	***p***	***β***	**Lower**	**Upper**	**VIF**	**Tolerance**
Intercept^a^	−17.31	1.48	−11.71	< 0.001					
Education years	0.07	0.08	0.89	0.37	0.03	−0.03	0.08	1.01	0.99
Age	0.30	0.02	12.88	< 0.001	0.45	0.38	0.52	1.46	0.69
PSS	0.08	0.04	2.32	0.02	0.08	0.01	0.14	1.32	0.76
PSQI	0.12	0.07	1.84	0.07	0.06	0.00	0.13	1.28	0.78
LEC	0.12	0.07	1.66	0.10	0.05	−0.01	0.11	1.09	0.92
Opioid years	0.07	0.03	2.59	0.01	0.09	0.02	0.16	1.47	0.68
Cocaine years	0.13	0.04	3.49	< 0.001	0.12	0.05	0.19	1.40	0.71
Cannabis years	−0.01	0.04	−0.29	0.77	−0.01	−0.07	0.06	1.29	0.77
Alcohol years	−0.01	0.03	−0.29	0.77	−0.01	−0.08	0.06	1.34	0.75
Race—White									
Black	1.12	0.51	2.19	0.03	0.15	0.02	0.28	1.04	0.96
Gender—Female									
Male	0.47	0.47	1.00	0.32	0.06	−0.06	0.19	1.11	0.91

Despite the absence of collinearity problems in the “years of regular drug use” model, we wanted to remove the contribution of the inevitable relationship between years of drug use and age (age was a covariate in the “years of regular drug use” model). Therefore, we fit a second model in which the main predictor was years of regular drug use as a *proportion* of age. Age was not used as a separate covariate. The overall model fit was significant [*F*_(10,740)_ = 20.80, *p* < 0.001] with adjusted *R*^2^ = 0.21. Greater allostatic load was associated with greater lifetime proportion of years using opioids (β = 0.22, 95%*CI* [0.14, 0.29]), cocaine (β = 0.22, 95%*CI* [0.15, 0.29]), or alcohol (β = 0.09, 95%CI [0.02, 0.16]), smaller cannabis use proportions (β = −0.09, 95%CI [−0.16, −0.02]), Black race (β = 0.28, 95%*CI* [0.13, 0.43]), male sex (β = 0.14, 95%*CI* [0.00, 0.28]), and poorer sleep quality (β = 0.10, 95%*CI* [0.03, 0.17]). Variance inflation factors for all covariates were less than or equal to 1.32, indicating no problem with collinearity. A full summary for the “lifetime proportion of drug use” model is presented in Table 5.

**Table 5:** Summary for a multiple linear regression that considers allostatic load as a function of life proportion spent using drugs as calculated from addiction severity index responses.

	**Overall model test**								
	***R***	***R^**2**^***	***Adj R^**2**^***	***F***	***df1***	***df2***	***p***		
Proportion model	0.47	0.22	0.21	20.80	10	740	< 0.001		
						**95% CI**		**Collinearity**	
**Predictor**	***B***	***SE***	***t***	***p***	***β***	**Lower**	**Upper**	**VIF**	**Tolerance**
Intercept^a^	−8.90	1.46	−6.10	< 0.001					
Education	0.13	0.08	1.51	0.13	0.05	−0.01	0.11	1.01	0.99
PSS	0.06	0.04	1.59	0.11	0.06	−0.01	0.13	1.32	0.76
PSQI	0.20	0.07	2.68	0.01	0.10	0.03	0.17	1.28	0.78
LEC	0.12	0.08	1.49	0.14	0.05	−0.02	0.12	1.10	0.91
Opioid P	7.89	1.34	5.90	< 0.001	0.22	0.14	0.29	1.27	0.79
Cocaine P	11.17	1.85	6.02	< 0.001	0.22	0.15	0.29	1.26	0.80
Cannabis P	−4.17	1.59	−2.62	0.01	−0.09	−0.16	−0.02	1.22	0.82
Alcohol P	3.85	1.49	2.58	0.01	0.09	0.02	0.16	1.20	0.84
Race—White									
Black	2.08	0.57	3.69	< 0.001	0.28	0.13	0.43	1.03	0.97
Gender—Female									
Male	1.04	0.53	1.96	0.05	0.14	0.00	0.28	1.12	0.90

## Study 2 Method

The epidemiologic sample in these analyses was from the National Health and Nutrition Examination Survey (NHANES), a large-scale program of data collection implemented by the Centers for Disease Control (CDC) to better understand the intersections of health, nutrition, and other aspects of life in a sample representative of the United States population. NHANES cohorts participated in 2-year cycles, and these cohorts may be combined to form larger participant pools, a practice that is especially useful when examining an infrequently reported behavior like illicit drug use. Because only a subset of the NHANES participants complete the measures needed for analyses of drug use and allostatic load, we chose to sample from four cohorts collected between 2009 and 2016, mimicking the data collection period from our Study 1. A sample-size breakdown is shown in Table 6.

**Table 6:** Participants in 2009–2016 NHANES cohort, broken down by NHANES cycle and study subsample.

***N***	**2009–2010**	**2011–2012**	**2013–2014**	**2015–2016**	**Total**
Screened	13,272	13,431	14,332	15,327	56,362
Interviewed	10,537	9,756	10,175	9,971	40,439
Examined	10,253	9,338	9,813	9,544	38,948
Drug use survey	3,747	3,344	3,701	3,428	14,220
Fasting subsample	3,297	2,821	2,967	2,764	11,849
Drug use sample and fasting subsample	2,212	2,015	2,166	2,019	8,412

### Allostatic Load Measurement

For these analyses, we constructed allostatic-load scores using the dichotomization approach described in the introduction. We decided against running CCAs on the NHANES data because combining four cohorts (a useful move in terms of sample size) affects measurement concordance such that the CCA would not have been similar to that of Study 1. An additional consideration was that the dichotomization approach has been used in almost every prior published analysis of allostatic load in NHANES data (18) (In sensitivity analyses reported in the Supplementary Material, we also reanalyzed the main results from study 1 using the same dichotomization approach as in study 2).

In an effort to keep variables consistent between the two studies, we used the following measures for allostatic load: SBp, DBp, resting heart rate, total cholesterol, high density lipoprotein, glycohemoglobin (HbA1c), urine albumin, urine creatinine, BMI, and waist circumference. We were unable to include CrP and DHEAs, as these were missing from one or more of the NHANES cohorts. Individual risk zones were defined by clinically relevant risk indications for each measure, using the studies surveyed in Duong et al.'s (18) review of NHANES allostatic load studies (49, 67, 68); they are summarized in the Supplementary Material. Data points falling within the defined risk zones were assigned a value of 1; all others were assigned a value of 0; the values were then summed into the composite allostatic load score. Because NHANES 2009–2016 collection procedure for albumin and creatinine did not concord with previous studies using clinically relevant cutoff values, we used the uppermost quartile as the designated risk-zone. Participants currently taking medication to manage hypertension and/or high serum cholesterol, as indicated by questionnaire data, were assigned a positive risk indication for SBp and/or total cholesterol if their lab values fell within clinically normative range.

### Covariates

Sociodemographic covariates were consistent with those we used in Study 1: age as a continuous variable, biological sex (male, female), race/ethnicity (recoded as Black, White, or Hispanic), and education level (no high school, some high school, high school graduate, some college, college graduate). Depression symptoms were included as assessed by the PHQ-9, a questionnaire based on DSM-IV criteria. Lifetime minor medical conditions were included as a proxy measure of accumulated physiological stress and were assessed by summing positive responses to items on the medical-history questionnaire. Although the NHANES does not include a measure like the Pittsburgh Sleep Quality Index, it does ask participants to report their average hours of sleep per night; we included those as a continuous covariate. Other elements of the NHANES data were considered for use as covariates to reflect physical functioning, physical activity, diet behavior, and occupation, but the measures were too inconsistent across cohorts or were not administered to enough participants.

Drug-use variables were created from responses to NHANES surveys on alcohol drinking, cigarette smoking, and use of other drugs. For alcohol, participants reported the number of times they drank and the number of drinks they consumed on average within a particular time frame over the past 12 months. They had the option to specify whether they had drunk alcohol “x” number of times over the past weeks, months, or years. Dummy variables were created to standardize all use reports to a “drinks per year” estimate. Binge drinking was assessed by participants' response to a single item covering the past year. For tobacco, participant responses to cigarettes smoked per period of time were standardized to “cigarettes per year” estimate. Coding the use of other drugs presented a unique challenge, as items on the DUQ were inconsistent between drugs, impeding uniformity in operationalization. First, to mirror the operationalization from Study 1, we calculated a years of use variable for cannabis, cocaine, and heroin from the difference between reported age of initiation and time since last used. Because the DUQ does not ask participants whether the intervening time between initiation and last use involved regular use, we also sought to calculate estimates of drug use amount for a separate model. This posed additional challenges, as the DUQ asks participants to report how often per month they used cannabis as a range on a Likert scale, but asks for the value precisely for cocaine, heroin, and methamphetamine. Additionally, the DUQ asks participants to estimate lifetime number of uses of cocaine and methamphetamine, but not cannabis or heroin. We summed responses to the lifetime use Likert-item ratings (1 = once, 2 = 2–5 times, 3 = 6–19 times, 4 = 20–49 times, 5 = 50–99 times, 6 = 100 times or more) for cocaine (DUQ272), methamphetamine (DUQ352), and all injection drugs (DUQ410) into a single item on illicit drug use. Lastly, because the NHANES drug-use questionnaire appeared better suited to dichotomous coding of drug-use histories, and due to lack of measurement concordance for substance amount, we also coded drug use dichotomously. Descriptive data are reported in Table 7 for all continuous measures. Proportions of participants within each factor variable are located in the Supplementary Material.

**Table 7:** Means and standard deviation for all continuous NHANES variables.

	**Black**				**Hispanic**				**White**			
	**Female**	**Male**		**Female**		**Male**		**Female**			**Male**	
	***M***	**σ**	***M***	**σ**	***M***	**σ**	***M***	**σ**	***M***	**σ**	***M***	**σ**
Age	42.60	15.50	44.30	16.10	43.00	15.50	41.90	15.60	42.90	15.00	42.30	14.90
Allostatic load	4.15	2.62	4.48	2.67	3.53	2.59	4.46	2.81	3.12	2.59	3.90	2.70
PHQ-9 score	4.17	5.20	2.80	4.14	4.20	5.18	2.91	4.44	3.93	4.87	3.07	4.56
Lifetime medical conditions	1.51	1.49	1.06	1.37	1.24	1.39	0.82	1.20	1.41	1.58	1.08	1.38
Drinks/Year	123	335	256	470	71	186	218	415	121	251	247	416
Cigarettes/Year	1,186	2,692	2,012	3,359	655	1,872	1,762	3,698	1,933	3,514	2,868	4,600
Illicit drug use amount	1.00	2.36	1.85	2.94	0.59	2.12	1.50	3.38	1.22	2.87	2.27	4.10
Average sleep hours	6.79	1.66	6.84	1.68	7.16	1.54	7.12	1.49	7.20	1.43	7.04	1.40
Cannabis years	4.35	8.64	7.37	11.20	1.54	5.29	3.16	7.25	3.88	18.70	6.42	10.40
Cocaine years	0.87	4.24	1.68	5.86	0.33	2.12	1.30	4.68	0.72	3.31	1.64	5.15
Heroin years	0.16	1.77	0.35	2.92	0.02	0.42	0.11	1.10	0.33	16.50	0.25	15.80

### Statistical Analyses

We conducted all analyses using R version 3.6.1 and Jamovi version 1.1.9. Response bias on questionnaires was evaluated according to a “10% missing” rule suggested by NHANES analysis guidelines. The R package {survey} was designed to handle the necessities imposed by the NHANES probability-proportional-to-size survey (PPS) design. Sampling error was calculated using Taylor-series linearization in an automated function. By passing masked variance units, masked pseudo stratum, and sampling weights to the package function “svydesign,” we created a referenceable set of sample parameters that were subsequently passed to all analyses. Additionally, NHANES publishes survey weights for all individual cohorts and relevant subsamples. Proper survey weights for the smallest analyzable group, the fasting-subsample, were adjusted according to NHANES data analysis recommendations (i.e., dividing the fasting subsample weight, “wtsaf2yr,” by 4) to account for the use of an 8-year cohort prior to inclusion in the survey-design item. Degrees of freedom for regression models were determined by the number of sampling units and strata containing the referenced variables, such that subtracting the number of strata from the number of sampling units resulted in residual degrees of freedom.

To replicate findings from Study 1, we implemented a general linear regression model that considered drug use as the number of years between age of initiation to a drug and age when last used the drug. We checked for collinearity problems by using a correlation matrix, coefficient covariance matrix, and variable VIFs, using R package {svydiags} and {jtools}. No collinearity problems were found, and VIFs did not exceed 1.15 for any covariates.

From the recoding of continuous substance-use measures into dichotomous factors, we fit a third general linear regression model that considers allostatic load a function of those substance-use dichotomies. For all models, covariates were entered simultaneously, and non-significant covariates were not trimmed from the models.

## Study 2 Results

Using the NHANES data, we fit a general linear regression model in which sought to replicate findings from Study 1 using an “estimated years of use” variable for cannabis, heroin, and cocaine, created by taking the difference between age of initiation and age when last used. The model intercept was significant (*t* = 5.17, *p* < 0.01) with adjusted *R*^2^ = 0.20. Greater allostatic load was associated with older age (β = 0.23, 95%*CI* [0.21, 0.25]), male sex (β = 0.33, 95%*CI* [0.29, 0.37]), Hispanic ethnicity (β = 0.16, 95%*CI* [0.11, 0.21]), Black race (β = 0.23, 95%*CI* [0.18, 0.28]), all education levels prior to college graduate (see Table 8), household yearly incomes below $99,999 (see Table 8), more minor medical conditions (β = 0.23, 95%*CI* [0.21, 0.25]), and higher PHQ-9 scores (β = 0.04, 95%*CI* [0.02, 0.06]). Cannabis, cocaine, and heroin year variables were not significant predictors of allostatic load.

**Table 8:** Statistical outputs from the NHANES general linear regression model that considers allostatic load a function of years using cannabis, cocaine, and heroin and psycho-, physio-, socio-demographic covariates.

**NHANES years model**						**95% CI**		**Collinearity**	
	***B***	***SE***	***t***	***p***	**β**	**Lower**	**Upper**	**VIF**	**Tolerance**
Intercept^a^	1.27	0.25	5.17	< 0.001					
Age	0.04	0.00	21.12	< 0.001	0.23	0.21	0.25	1.10	0.91
Male gender	0.90	0.05	16.51	< 0.001	0.33	0.29	0.37	1.03	0.97
Race—White								1.06	0.95
Black	0.63	0.07	9.09	< 0.001	0.23	0.18	0.28		
Hispanic	0.42	0.07	6.21	< 0.001	0.16	0.11	0.21		
Education Level—College grad								1.05	0.95
HS grad	0.55	0.08	6.73	< 0.001	0.20	0.14	0.26		
No HS	0.49	0.12	4.12	< 0.001	0.18	0.10	0.27		
Some college	0.57	0.07	7.75	< 0.001	0.21	0.16	0.26		
Some HS	0.57	0.10	5.99	< 0.001	0.21	0.14	0.28		
Household income—over 100k								1.04	0.96
20,000–54,999	0.31	0.08	3.78	< 0.001	0.12	0.06	0.18		
55,000–99,999	0.28	0.09	3.10	0.00	0.10	0.04	0.17		
<20,000	0.20	0.10	2.09	0.04	0.07	0.00	0.14		
Lifetime medical conditions	0.43	0.02	20.49	< 0.001	0.23	0.21	0.25	1.14	0.88
PHQ-9 score	0.02	0.01	3.99	< 0.001	0.04	0.02	0.06	1.07	0.93
Average sleep hours	−0.02	0.02	−0.89	0.37	−0.01	−0.03	0.01	1.01	0.99
Cannabis years	0.00	0.00	0.67	0.50	0.01	−0.01	0.03	1.09	0.92
Cocaine years	0.00	0.01	0.35	0.73	0.00	−0.02	0.02	1.09	0.92
Heroin years	0.00	0.00	0.79	0.43	0.01	−0.01	0.03	1.00	1.00
*N*	8371								
Residual degrees of freedom	46								
*R* ^2^	0.198								
Adjusted *R*^2^	0.196								

Then, to examine the relationship between substance-use-*amount* estimates and allostatic load, we implemented a general linear regression model that coded drug-use variables as continuous amounts, scored to represent the volume of a drug or drug class used. The model intercept was significant (*t* = 5.14, *p* < 0.01) with adjusted *R*^2^ = 0.20. Quantified past-year amount estimates were not significant predictors of allostatic load, but the same significant relationships were observed for the sociodemographic predictors as in the “years of use” model, such that model statistics were nearly identical across the two models; see the Supplementary Material for more detail.

Lastly, we fit a general linear regression model that used dichotomized substance-use variables as predictors of allostatic load. The model intercept was significant (*t* = 5.45, *p* < 0.01) with adjusted *R*^2^ = 0.20. As in the previous two models, all sociodemographic factors were significant predictors of allostatic load (see Table 9), but so were past-year alcohol binge drinking (β = 0.02, 95%*CI* [0.003, 0.04]) and regular cannabis use (β = −0.02, 95%*CI* [−0.04, −0.00]), although both standardized β effect sizes were much closer to zero than to those of other significant predictors in the model.

**Table 9:** Statistical outputs from the NHANES general linear regression model that considers allostatic load a function of substance use dichotomies and psycho-, physio-, socio-demographic covariates.

**NHANES groups model**						** 95% CI**		**Collinearity**	
	***B***	***SE***	***t***	***p***	**β**	**Lower**	**Upper**	**VIF**	**Tolerance**
Intercept^a^	1.02	0.15	34.71	< 0.001					
Age	0.04	0.00	20.10	< 0.001	0.23	0.20	0.25	1.15	0.87
Male gender	0.91	0.06	16.38	< 0.001	0.34	0.30	0.38	1.05	0.95
Race—White								1.06	0.94
Black	0.63	0.07	9.05	< 0.001	0.23	0.18	0.28		
Hispanic	0.39	0.07	5.70	< 0.001	0.15	0.10	0.20		
Education level—college Grad								1.05	0.95
HS Grad	0.57	0.08	6.85	< 0.001	0.21	0.15	0.27		
No HS	0.48	0.12	3.97	< 0.001	0.18	0.09	0.26		
Some college	0.59	0.07	7.92	< 0.001	0.22	0.16	0.27		
Some HS	0.59	0.10	6.04	< 0.001	0.22	0.15	0.29		
Household income—over 100k								1.04	0.96
20,000–54,999	0.31	0.08	3.78	< 0.001	0.12	0.06	0.18		
55,000–99,999	0.27	0.09	3.08	0.00	0.10	0.04	0.17		
< 20,000	0.20	0.10	2.10	0.04	0.07	0.00	0.14		
Lifetime medical conditions	0.44	0.02	20.58	< 0.001	0.23	0.21	0.25	1.15	0.87
PHQ-9 score	0.03	0.01	4.21	< 0.001	0.04	0.02	0.07	1.08	0.93
Average sleep hours	−0.02	0.02	−0.93	0.36	−0.01	−0.03	0.01	1.01	0.99
Regular tobacco use	−0.01	0.06	−0.18	0.86	0.00	−0.02	0.02	1.14	0.87
Ever use illicit drugs	−0.07	0.08	−0.84	0.40	−0.01	−0.03	0.01	1.11	0.90
Past year alcohol binge	0.20	0.09	2.25	0.03	0.02	0.00	0.04	1.04	0.96
Regular cannabis	−0.17	0.08	−2.05	0.04	−0.02	−0.04	0.00	1.14	0.88
*N*	8371								
Residual degrees of freedom	45								
*R* ^2^	0.199								
Adjusted *R*^2^	0.197								

## Sensitivity Analysis

To help ensure that differences between findings in Study 1 and Study 2 were not solely attributable to a difference in allostatic load scoring method (CCA vs. dichotomization), we reanalyzed the data from Study 1 according to the same dichotomization method used in Study 2. Doing so resulted in poorer model fit indices for both the “years” and “proportion” multiple-regression models but did not alter the effects observed for opioid-use history or cocaine-use history. Full results are shown in the Supplementary Material.

## Discussion

The purpose of these two sets of secondary analyses was to determine whether self-reported drug-use histories would be associated with higher allostatic load, after controlling for likely confounds. Our use of two different samples allowed us to examine the importance of context and specificity to the allostatic load paradigm.

### Demographic Characteristics' Influence on Allostatic Load

In Study 1 using multiple regression, we found that age, Black race, and male sex were all significant predictors of allostatic-load scores. These findings were supported by the nationally representative NHANES data we analyzed in Study 2, providing some assurance that demographic effects in Study 1 were not only artifacts of canonical overfitting. In the NHANES data, but not our clinic data, lower education level was associated with allostatic load; this discrepancy was probably due to insufficient variation in education in our clinical sample. Similarly, lower household incomes (<$99,999 USD vs. over $100,000 USD) were associated with increased allostatic load in the NHANES sample. Considering results from both studies, there were protective effects of White race, female sex, and higher attained education levels. These findings are not novel, but do add to the literature on likely health effects of racial discrimination through various psychological and physiological mechanisms (33, 35–38, 40, 42, 68).

### Psychometric, Sleep Quality, and Health Factors' Influence on Allostatic Load

Allostatic-load measures have usually shown associations not only with demographics, but also with psychometric measures, health behaviors, and lifetime medical histories, and our findings reflect that. In Study 1, higher PSS ratings in the “years” model and poor reported sleep quality in the “proportion” model emerged as predictors of increased allostatic load. The effect of perceived stress, as measured by standardized regression β, was similar in magnitude to that of both opioid-use years and cocaine-use years in that model, whereas sleep quality measured by PSQI's effect was less than half the size of cocaine-use proportion or heroin-use proportion and similar in size to that of alcohol-use proportion and marijuana-use proportion. Chronic experiential stress is thought to have a robust effect on overall health, and because this type of stress activates allostasis mechanisms, it is not surprising that a significant effect was observed for ratings of perceived stress in Study 1 and for ratings of depression symptoms in Study 2.

Poor sleep quality has also been previously associated with a myriad of negative health outcomes (49, 50). Although average sleep hours were not a robust predictor of allostatic load in the NHANES data, PSQI scores were an important covariate in our clinical sample. Sleep quality may be especially important to those with a history of illicit drug use, as opioids and illicit stimulants negatively influence sleep architecture (69, 70). A history of poor sleep may be inherent in our “years of drug use” measure, making it an important factor in the development of increased allostatic load in those with longer histories of substance use.

Although traumatic life events, as scored by the LEC, did not reach statistical significance in either model from Study 1, the 95% confidence intervals for its coefficient crossed zero only narrowly. The LEC may not have been the most sensitive measure for our purposes, as studies that found an association between previous life trauma and allostatic load have generally employed measures specific to childhood like the Adverse Childhood Experiences (ACE) questionnaire (25, 27, 29, 71). In Study 2, depression symptoms (assessed by the PHQ-9) and lifetime minor medical conditions were both significant predictors of increased allostatic load. As with perceived stress, these findings are consistent with previous findings that mood-related disorders can have a negative impact on overall health and on allostatic load (47, 72, 73). We fully expected lifetime medical conditions to predict allostatic load; we included that predictor in the model to help parse a potential effect of drug from what could otherwise have been a large confound. The decision appears to have been warranted, as lifetime medical conditions were the strongest predictor of allostatic load and were not significantly correlated or collinear with any of the drug-use measures from NHANES.

### Drug-Use Histories and Allostatic Load

Study 1, in a clinical cohort with high rates of drug use and treatment seeking, showed that years spent using illicit opioids and cocaine did predict higher allostatic load, independent of participant age, race, sex, perceived stress, sleep quality, traumatic life events, and educational attainment. After we converted the “drug years” variables into “proportion of life spent using drugs” (an approach we thought might be more informative than simply controlling for age in the model), we found that higher allostatic load was associated with opioid use, cocaine use, and alcohol drinking. Opioid and cocaine use histories showed very similar effect sizes to the other when conceptualized as “years spent using” or “proportion of life spent using,” with cocaine use history being slightly stronger in both. Cannabis history, the most common form of drug use in the sample, did not significantly predict allostatic load in the “years of use, controlling for age” model—and had a small protective effect in the “proportion of life spent using” model and as a dichotomous “regular cannabis use” factor in the NHANES data. There are at least two potential mechanisms through which cannabis use could protect against increases in allostatic load. First, some data suggest that cannabis use can have opioid-sparing effects in people who use both to manage chronic pain, a prevalent condition in people who use opioids (74), though we should be cautious in our interpretation of protective cannabis effects, as findings are mixed and differ substantially by observed population. Second, cannabis use may protect against allostatic-load increases via anti-inflammatory properties, as posited by Lohr et al. (75) in their detailed discussion of how cannabis use may beneficially influence allostatic load markers.

The associations between allostatic load and drug-use history were smaller and not statistically significant in a nationally representative sample from the NHANES 2009–2016. Illicit drug use in our clinical sample showed effects that were smaller than those of age or race/ethnicity but larger than those of psychological stress and sleep quality, but all substance-use effect sizes were near zero in all three models using NHANES sample data. Detecting a signal of increased allostatic load may be particularly difficult using traditional biomarker operationalizations, as the effects from substance use itself may not modulate those biomarkers to clinically significant levels after acute drug effects have dissipated. Less traditional markers of allostatic load, such as changes in cortical structure, may be better suited for substance use signal detection than traditional allostatic load measures. This approach is becoming more feasible with large longitudinal neuroimaging studies (76).

Our findings from Study 1 are consistent with the theory that drug use damages allostasis systems and facilitates increased allostatic load in addiction, with the caveat that cross-sectional data cannot directly test a longitudinal hypothesis. Our CCA findings—though intended as a step in our methodology, not as a test of a hypothesis—suggest that demographic characteristics such as race show much stronger associations with raw allostatic load measures than do self-reported elements of psychosocial history, with the canonical coefficient for race being much larger (0.30) than that of perceived stress (0.02) or sleep quality (0.01).

When comparing study 1 to study 2, it is important to remember that there were substantial differences in sample composition. This was a strong test of whether our findings from a clinical sample would also apply to a US probability sample—and they generally did not. Our clinical sample, originally recruited for natural-history studies of either substance use or SUD treatment, included a substantial number of people with extensive histories of substance, far exceeding observed U.S. population prevalence for cocaine and opioid use in particular. In NHANES, a much more heterogeneous and nationally representative sample designed to study the health status of U.S. adults generally, any substance-use-related increase in allostatic load may have been too difficult to detect among the myriad sources of variation in allostatic load measures.

Aside from the aforementioned issues with measure concordance and drug-use operationalizations in the combined NHANES cohorts, the discrepancy between results from our two sets of analyses can be contextualized around the extreme societal stigma associated with SUDs. In his 2005 paper, Robin Room (77) notes that those most affected by SUDs experience many forms of social exclusion that can prevent them from societal integration. This being the case, general-population surveys may fail to capture the experiences of the subset of drug users who have an SUD, especially those previously or currently involved with the criminal justice system. On the other hand, data collected from outpatient treatment clinics may better represent the reality of those with long histories using illicit substances.

## Limitations

Drug use does not occur in isolation. Factors like housing insecurity, financial stress, and legal problems often accompany heavy use of psychoactive drugs, and, without time-series data, we cannot disentangle drug effects on allostatic load from those of other stressors. Because this was a secondary analysis of cross-sectional data, we can only conclude that people with lengthier histories of opioid and cocaine use present with higher allostatic load. While it may be the case that years of drug use led to the development of increased allostatic load, we could not control for the plausible possibility that people in our sample with higher allostatic load, before initiating drug use, tended to use drugs for lengthier periods of time prior to enrolling in our study. Both explanations are compatible with an allostasis model of addiction, as repeated drug use leading to addiction can accelerate allostatic load, and people with increased allostatic load may have a higher risk of developing addiction.

Another limitation, especially relevant to those who use substances, is the lack of data on our clinical sample participants' tobacco-use history. In a recent systematic review on health behaviors and allostatic load, Suvarna et al. (78) discussed connections between tobacco smoking and increased allostatic load. We could not assess, or control for, that possibility in clinical sample models, though we did not observe a significant relationship between cigarette smoking and allostatic load while controlling for other substance use and demographic factors in NHANES models.

A possible source of bias in the data is differential mortality as a function of drug class, especially for people who meet criteria for an SUD; for example, early mortality in people with OUD was documented in longitudinal work (79) even before the current fentanyl-exacerbated crisis of overdose deaths. Early mortality could effectively right-censor some of the data: people who die young would not be able to express the allostatic load that would have accumulated if they had lived. This would tend to blunt the associations for which we were testing.

Generalizability of Study 1 results is hindered by the heavy demographic skew in our sample. By including sociodemographic variables as covariates in our analyses, we attempted to control the influence of these factors on our results. Nonetheless, results may be difficult to replicate in populations that have heavily differing characteristics, as evidenced by the null results from NHANES 2009–2016. Failure to replicate findings from the clinical sample may be attributable to limitations inherent in the NHANES data. The NHANES DUQ does not ask participants whether they regularly used a substance in the period between initiation and most recent use, and consequently we must consider this assumption as a limitation in our calculation of substance use histories. Additionally, combining multiple NHANES cohorts to increase the analyzable sample size hindered our use of CCA allostatic load scoring paradigm, and our use of the upper quartile risk dichotomization in those data may have hindered our ability to detect a signal.

## Future Directions

Longitudinal evidence is needed in order to establish evidence for a dose-response relationship between illicit drug use and allostatic load. By collecting baseline measures of current allostatic load and history of drug use, researchers could determine the degree of change in these values relative to participants' ongoing drug use in a more refined fashion. Drug-use indices were rather broad in our analyses, as we had to rely on participants' reports on the length of time they had spent regularly using particular substances. Future longitudinal studies could benefit greatly from attempting to quantify drug use by both amounts used and time spent using.

A longitudinal design would also provide much needed evidence, or lack thereof, for the observed directionality of the relationship between allostatic load and drug use. The relationship between traumatic life events and increased allostatic load is well-supported, and ongoing studies that collect data not only on substance use amount over time [e.g., (76)] but also the temporality of potentially traumatic life events could provide much-needed insights into sequencing and possible causation.

## Data Availability Statement

The raw data supporting the conclusions of this article will be made available by the authors, without undue reservation.

## Ethics Statement

The studies involving human participants were reviewed and approved by National Institute on Drug Abuse (NIDA) Institutional Review Board. The patients/participants provided their written informed consent to participate in this study.

## Author Contributions

KLP, KP, and DE conceived and planned the experiments. JS conceived the idea to include nationally representative data. JR conducted all analyses with supervision and mentorship from DE. JR led in drafting the initial manuscript, to which DE gave crucial feedback. All authors contributed to the interpretation of the results and development of the final manuscript.

## Conflict of Interest

The authors declare that the research was conducted in the absence of any commercial or financial relationships that could be construed as a potential conflict of interest.

## Publisher's Note

All claims expressed in this article are solely those of the authors and do not necessarily represent those of their affiliated organizations, or those of the publisher, the editors and the reviewers. Any product that may be evaluated in this article, or claim that may be made by its manufacturer, is not guaranteed or endorsed by the publisher.
