# Supplementary material for: Exploring the Relationship Between Substance Use and Allostatic Load in a Treatment/Research Cohort and in a US Probability Sample (NHANES 2009–2016)
Source: Front Psychiatry. 2021 Aug 2;12:630195. doi: 10.3389/fpsyt.2021.630195 (PMC8367194; doi:10.3389/fpsyt.2021.630195)
Supplement: Supplementary file 1 [file Data_Sheet_1.docx]

Supplementary Material

# Supplementary Tables.

## Frequency table with proportions for attained education level by race and gender in 2009-2016 NHANES

|  |  | Female | | Male | |
| --- | --- | --- | --- | --- | --- |
| Education Level | | *N* | *P* | *N* | *P* |
| No Highschool | Black | 18 | 0.02 | 20 | 0.02 |
|  | Hispanic | 270 | 0.23 | 265 | 0.23 |
|  | White | 49 | 0.02 | 65 | 0.03 |
| Some Highschool | Black | 126 | 0.13 | 158 | 0.18 |
|  | Hispanic | 217 | 0.18 | 235 | 0.20 |
|  | White | 193 | 0.09 | 235 | 0.11 |
| Highschool Graduate | Black | 229 | 0.24 | 282 | 0.32 |
|  | Hispanic | 221 | 0.19 | 255 | 0.22 |
|  | White | 380 | 0.18 | 459 | 0.21 |
| Some College | Black | 361 | 0.38 | 279 | 0.31 |
|  | Hispanic | 332 | 0.28 | 260 | 0.22 |
|  | White | 693 | 0.33 | 642 | 0.30 |
| College Graduate | Black | 222 | 0.23 | 147 | 0.17 |
|  | Hispanic | 143 | 0.12 | 142 | 0.12 |
|  | White | 775 | 0.37 | 733 | 0.34 |

## Frequency table with proportions for self-reported household income in NHANES 2009-2016

|  |  | Female | | Male | |
| --- | --- | --- | --- | --- | --- |
| Household Income | | *N* | *P* | *N* | *P* |
| Greater than 100K | White | 468 | 0.22 | 530 | 0.25 |
|  | Black | 88 | 0.09 | 111 | 0.13 |
|  | Hispanic | 100 | 0.08 | 97 | 0.08 |
| 55k to 99k | White | 473 | 0.23 | 487 | 0.23 |
|  | Black | 165 | 0.17 | 175 | 0.20 |
|  | Hispanic | 214 | 0.18 | 210 | 0.18 |
| 21k to 55k | White | 758 | 0.36 | 785 | 0.37 |
|  | Black | 449 | 0.47 | 404 | 0.46 |
|  | Hispanic | 592 | 0.50 | 613 | 0.53 |
| Below 20k | White | 393 | 0.19 | 333 | 0.16 |
|  | Black | 254 | 0.27 | 197 | 0.22 |
|  | Hispanic | 278 | 0.23 | 238 | 0.21 |

## Frequency table with proportions for drug use binomials in 2009-2016 NHANES

|  |  | Female | | Male | |
| --- | --- | --- | --- | --- | --- |
| Regular Tobacco |  | *N* | *P* | *N* | *P* |
| No | Black | 654 | 0.68 | 450 | 0.51 |
|  | Hispanic | 907 | 0.77 | 624 | 0.54 |
|  | White | 1238 | 0.59 | 1065 | 0.50 |
| Yes | Black | 302 | 0.32 | 437 | 0.49 |
|  | Hispanic | 277 | 0.23 | 534 | 0.46 |
|  | White | 854 | 0.41 | 1070 | 0.50 |
| Regular Cannabis |  |  |  |  |  |
| No | Black | 827 | 0.87 | 658 | 0.74 |
|  | Hispanic | 1110 | 0.94 | 1000 | 0.86 |
|  | White | 1818 | 0.87 | 1609 | 0.75 |
| Yes | Black | 129 | 0.13 | 229 | 0.26 |
|  | Hispanic | 74 | 0.06 | 158 | 0.14 |
|  | White | 274 | 0.13 | 526 | 0.25 |
| Alcohol Binge Behavior |  |  |  |  |  |
| No | Black | 892 | 0.93 | 792 | 0.89 |
|  | Hispanic | 1102 | 0.93 | 874 | 0.75 |
|  | White | 1979 | 0.95 | 1835 | 0.86 |
| Yes | Black | 64 | 0.07 | 95 | 0.11 |
|  | Hispanic | 82 | 0.07 | 284 | 0.25 |
|  | White | 113 | 0.05 | 300 | 0.14 |
| Used Illicit Meth / Cocaine / Heroin |  |  |  |  |  |
| No | Black | 861 | 0.90 | 703 | 0.79 |
|  | Hispanic | 1091 | 0.92 | 950 | 0.82 |
|  | White | 1806 | 0.86 | 1655 | 0.78 |
| Yes | Black | 95 | 0.10 | 184 | 0.21 |
|  | Hispanic | 93 | 0.08 | 208 | 0.18 |
|  | White | 286 | 0.14 | 480 | 0.22 |

## Clinically relevant cut-off values indicating the risk zones for each biomarker

| Biomarker | Cut Point Value |
| --- | --- |
| Albumin (g) | Uppermost Quartile |
| Creatinine (mg/dl) | Uppermost Quartile |
| Body Mass Index (kg/m^2^) | ≥ 30 |
| HDL Cholesterol (mg/dl) | < 40 |
| Total Cholesterol (mg/dl) | ≥ 240 |
| HbA1c (%) | ≥ 6.4 |
| Systolic Blood Pressure (mm Hg) | ≥ 140 |
| Diastolic Blood Pressure (mm Hg) | ≥ 90 |
| Resting Heart Rate (Bpm) | ≥ 90 |
| Waist Circumference - Men | ≥ 102.7 |
| Waist Circumference - Women | ≥ 89.0 |

## Statistical outputs from the general linear regression model that considers allostatic load a function of quantified drug use amount and psycho-, physio-, socio-demographic covariates

| Estimated Amount Model |  |  |  |  |  | 95% *CI* | | Collinearity | |
| --- | --- | --- | --- | --- | --- | --- | --- | --- | --- |
| Predictor | *B* | *SE* | *t* | *p* | *β* | Lower | Upper | VIF | Tolerance |
| Intercept ᵃ | 1.25 | 0.24 | 5.14 | < .001 |  |  |  |  |  |
| Age | 0.04 | 0.00 | 20.50 | < .001 | 0.23 | 0.21 | 0.25 | 1.13 | 0.89 |
| Male Gender | 0.93 | 0.06 | 16.62 | < .001 | 0.34 | 0.30 | 0.38 | 1.05 | 0.95 |
| Race – White ^a^ |  |  |  |  |  |  |  | 1.07 | 0.94 |
| Black | 0.63 | 0.07 | 8.96 | < .001 | 0.23 | 0.18 | 0.28 |  |  |
| Hispanic | 0.42 | 0.07 | 5.98 | < .001 | 0.15 | 0.10 | 0.20 |  |  |
| Education Level - College Grad ^a^ |  |  |  |  |  |  |  | 1.05 | 0.95 |
| HS Grad | 0.57 | 0.08 | 6.85 | < .001 | 0.21 | 0.15 | 0.27 |  |  |
| No HS | 0.51 | 0.12 | 4.28 | < .001 | 0.19 | 0.10 | 0.28 |  |  |
| Some College | 0.59 | 0.07 | 7.93 | < .001 | 0.22 | 0.16 | 0.27 |  |  |
| Some HS | 0.59 | 0.10 | 6.13 | < .001 | 0.22 | 0.15 | 0.29 |  |  |
| Household Income - over 100k ^a^ |  |  |  |  |  |  |  | 1.04 | 0.96 |
| 20,000 - 54,999 | 0.30 | 0.08 | 3.67 | < .001 | 0.11 | 0.05 | 0.17 |  |  |
| 55,000 - 99,999 | 0.27 | 0.09 | 2.97 | 0.00 | 0.10 | 0.03 | 0.16 |  |  |
| < 20,000 | 0.20 | 0.10 | 2.08 | 0.04 | 0.07 | 0.00 | 0.14 |  |  |
| Lifetime Medical Conditions | 0.43 | 0.02 | 20.29 | < .001 | 0.23 | 0.21 | 0.25 | 1.15 | 0.87 |
| PHQ-9 Score | 0.03 | 0.01 | 4.31 | < .001 | 0.05 | 0.02 | 0.07 | 1.08 | 0.93 |
| Average Sleep Hours | -0.02 | 0.02 | -0.90 | 0.37 | -0.01 | -0.03 | 0.01 | 1.01 | 0.99 |
| Drinks / Year | 0.00 | 0.00 | -1.32 | 0.19 | -0.01 | -0.03 | 0.01 | 1.05 | 0.96 |
| Lifetime Illicit Drug Amount | -0.01 | 0.01 | -0.86 | 0.39 | -0.01 | -0.03 | 0.01 | 1.08 | 0.93 |
| Cigarettes / Year | 0.00 | 0.00 | 0.28 | 0.78 | 0.00 | -0.02 | 0.02 | 1.10 | 0.91 |
| ᵃ Represents reference level |  |  |  |  |  |  |  |  |  |
|  |  |  |  |  |  |  |  |  |  |
| *N* | 8332 |  |  |  |  |  |  |  |  |
| Residual Degrees of Freedom | 46 |  |  |  |  |  |  |  |  |
| *R^2^* | 0.199 |  |  |  |  |  |  |  |  |
| Adjusted *R^2^* | 0.197 |  |  |  |  |  |  |  |  |

## Summary for a multiple linear regression that considers allostatic load as a function of life proportion spent using drugs as calculated from addiction severity index responses: Sensitivity analysis with allostatic load being scored using the same scoring paradigm as in NHANES 2009-2016

| Overall Model Test | | | | | | | |  |  |
| --- | --- | --- | --- | --- | --- | --- | --- | --- | --- |
| Proportion Model | *R* | *R²* | *Adj R²* | *F* | *df1* | *df2* | *p* |  |  |
|  | 0.34 | 0.12 | 0.11 | 9.91 | 10 | 740 | < .001 |  |  |
|  |  |  |  |  |  |  |  |  |  |
|  |  |  |  |  |  | *95% CI* | | Collinearity | |
| Predictor | *B* | *SE* | *t* | *p* | *β* | Lower | Upper | VIF | Tolerance |
| Intercept ᵃ | 0.13 | 0.49 | 0.27 | 0.79 |  |  |  |  |  |
| Education | 0.06 | 0.03 | 2.23 | 0.03 | 0.08 | 0.01 | 0.15 | 1.01 | 0.99 |
| PSS | 0.02 | 0.01 | 1.41 | 0.16 | 0.06 | -0.02 | 0.13 | 1.32 | 0.76 |
| PSQI | 0.05 | 0.02 | 2.17 | 0.03 | 0.08 | 0.01 | 0.16 | 1.28 | 0.78 |
| LEC | 0.02 | 0.03 | 0.67 | 0.50 | 0.02 | -0.05 | 0.10 | 1.10 | 0.91 |
| Opioid P | 1.63 | 0.45 | 3.65 | < .001 | 0.14 | 0.07 | 0.22 | 1.27 | 0.79 |
| Cocaine P | 3.25 | 0.62 | 5.22 | < .001 | 0.20 | 0.13 | 0.28 | 1.26 | 0.80 |
| Cannabis P | -0.87 | 0.53 | -1.62 | 0.11 | -0.06 | -0.14 | 0.01 | 1.22 | 0.82 |
| Alcohol P | 0.42 | 0.50 | 0.84 | 0.40 | 0.03 | -0.04 | 0.11 | 1.20 | 0.84 |
| Race: |  |  |  |  |  |  |  | 1.03 | 0.97 |
| Black - White | 0.25 | 0.19 | 1.30 | 0.20 | 0.10 | -0.05 | 0.26 |  |  |
| Gender: |  |  |  |  |  |  |  | 1.12 | 0.90 |
| Female - Male | -0.27 | 0.18 | -1.53 | 0.13 | -0.12 | -0.26 | 0.03 |  |  |
